# Psychiatric Comorbidity and Its Impact on Mortality in Patients Who Attempted Suicide by Paraquat Poisoning during 2000–2010

**DOI:** 10.1371/journal.pone.0112160

**Published:** 2014-11-11

**Authors:** Chemin Lin, Tzung-Hai Yen, Yeong-Yuh Juang, Ja-Liang Lin, Shwu-Hua Lee

**Affiliations:** 1 Department of Psychiatry, Chang Gung Memorial Hospital, Keelung branch, Keelung, Taiwan; 2 Department of Psychiatry, Chang Gung Memorial Hospital, Linkou branch, Taoyuan, Taiwan; 3 Department of Nephrology and Division of Clinical Toxicology, Chang Gung Memorial Hospital, Taipei, Taiwan; 4 School of Medicine, Chang Gung University, Taoyuan, Taiwan; University of Vienna, Austria

## Abstract

**Background:**

Paraquat poisoning is a lethal method of suicide used around the world. Although restricting its accessibility had been widely discussed, the underlying psychopathological mechanism of paraquat self-poisoning and its association with mortality have not yet been explicitly evaluated.

**Methods:**

We included all patients admitted to a tertiary general hospital in Taiwan between 2000 and 2010 following a suicide attempt by paraquat self-administration. Diagnoses were made upon psychiatric consultation based on the *Diagnostic and Statistical Manual of Mental Disorders, Fourth Edition (DSM-IV)* criteria. The risk of mortality was calculated by logistic regression with various psychiatric or medical covariates.

**Results:**

The consultation-liaison psychiatry team assessed 157 patients who attempted suicide by paraquat poisoning. Mood disorders (54.0%), including dysthymic (26.7%) and major depressive disorders (24.7%), were the most common psychiatric diagnoses among the self-poisoning patients. Among those who attempted suicide, 87 patients (58.0%) died and dysthymic disorder (OR = 5.58, 95% CI: 1.13–27.69; p<0.05) significantly increased the mortality risk after adjustment for relevant medical variables, including age, gender, severity index of paraquat poisoning (SIPP), and risk for respiratory failure.

**Conclusions:**

Awareness of comorbid psychiatric illnesses, especially dysthymic disorder, is vital in the prevention and treatment of suicide by paraquat poisoning.

## Introduction

Pesticide ingestion is a highly lethal method of suicide, especially in rural areas [1], and may account for up to one third of overall suicide deaths around the globe [2], [3]. Among all pesticide agents, paraquat is one of the most lethal [2] and is the most frequently used for suicide in Trinidad [4] and South Korea [5]. Paraquat intoxication accounts for two thirds of all pesticide suicides in Taiwan [6] and for a large proportion of suicides in other Asian countries [7]–[9]. Despite all efforts to ameliorate paraquat-induced toxicity through treatment [10], [11], the mortality rate of paraquat poisoning remains at approximately 60% [12], highlighting the importance of prevention. Limiting the availability [12], [13] or toxicity [14] of paraquat is an effective prevention strategy discussed by most previous epidemiological studies. Awareness and treatment of the underlying mental illness is another strategy that is discussed less frequently but underpins the critical role of the psychiatrist. Thus, this study aims to use face-to-face diagnostic interviews to explore comorbid psychiatric illness among all patients admitted following suicide attempts by paraquat self-poisoning. In light of the high mortality rate of attempted suicide by paraquat poisoning, we examined the association of psychiatric illness and the risk of mortality.

## Methods

This study complied with the guidelines of the Declaration of Helsinki and was approved by the Medical Ethics Committee of Chang Gung Memorial Hospital. Being a retrospective study, the Institutional Review Board approved and waived the need for specific informed consent from the patients. However, all necessary informed consent of acute organophosphate poisoning risk and all treatment modalities (including cardiopulmonary cerebral resuscitation, etc.) were obtained from all patients during their admission. All primary data were gathered according to the Strengthening the Reporting of Observational Studies in Epidemiology guidelines [15] and were analyzed anonymously by delinking the identifying information from the main data set.

The study hospital, Chang Gung Memorial Hospital, Linkou branch, is a 3700-bed tertiary referral medical center situated in northern Taiwan. All medical information was retrospectively collected from patients admitted to the ward of the department of nephrology following paraquat poisoning between December 2000 and December 2010. The doctors in emergency room (ER) would allocate all patients admitted after all kinds of poison intoxication, including paraquat, to the toxicology center in nephrology ward in our hospital, and this was our routine daily practice out of the contribution they made in the past several years [16]–[19]. This practice ensured no missed admitted paraquat intoxicated patients in our analysis. The collected data include laboratory examinations, amount of paraquat ingested, time elapsed between ingestion and hospital arrival, severity index of paraquat poisoning (SIPP; the product of plasma-paraquat level in milligrams per litre and time from paraquat intake to arrival in hours) [20], [21], pulse therapy regimen, and treatment outcome [22]. The inclusion/exclusion criteria and detoxification protocol (including gastric lavage, haemoperfusion and repeated pulse of methylprednisolone and cyclophosphamide with continuous dexamethasone therapy) for paraquat poisoning patients are described elsewhere [22]. In brief, we included patients aged 18 years or older admitted due to paraquat intoxication with detectable paraquat blood or urine level. Those exposed via dermal [23] or intravascular [24] route were excluded for fear of creating outliers in the data of blood paraquat level (too high in IV route, too low by dermal). In cooperation with nephrologists, the ascertainment of medical conditions [25] and the ascription of mortality to paraquat [10] could be undertaken during subsequent analysis. In addition, the psychiatric consultation sheets of patients who attempted suicide by paraquat poisoning were abstracted. A psychiatric consultation-liaison evaluation was required during admission if suicidal intent was suspected. The details of the consultation process have been elaborated in a previous article [26]. Briefly, we performed face-to-face interviews with the patient and a key informant, and collected collateral information (from medical or ambulance transport chart, and nursing staff) to ascertain the validity of suicidal intent and the reason for suicide. Diagnoses were made according to the *DSM-IV* criteria, and reasons for attempting suicide and recent life events were evaluated using the Chinese version of the Social Readjustment Rating Scale [27], [28]. We employed this scale as a check-list to categorize the reason for suicide and to weigh the underlying life stress. The scale is composed of 57 weighted stressful events with points ranging from 0 to 100. Death of children was given 95.9 points and was ranked highest. In cases with more than one reason for suicide, we registered the one with the highest rank. Sociodemographic data were retrieved from consultation records and included age, sex, marital status, education, and living condition. A special chart was recorded by the psychiatrist and reviewed by a senior professor in the weekly consultation-liaison service meeting to confirm the diagnosis and treatment recommendations.

### Statistical analysis

Continuous variables were expressed as means and standard deviations; and categorical variables as numbers and percentages. We used the chi-square or Fisher exact test for categorical variables and independent Student *t* tests for quantitative variables when comparing patients who ingested paraquat intentionally with those who ingested it accidentally. Univariate logistic regression analysis was used to examine risk factors of mortality. All medical factors related to mortalities were selected by thenephrologist based on clinical and existing knowledge. To further control for medical confounding factors, we performed a series of multivariate logistic regression analyses. In each model, we put age, gender, those medical variables significant (p<0.05) on univariate analysis along with each primary psychiatric diagnosis to examine its adjusted odds ratio associated with mortality. We used SPSS 16.0 software to perform the computations.

## Results

We identified 157 patients who were admitted after attempting suicide by paraquat poisoning during 2000 to 2010. The mean age, sex, marital status, employment status, whether living alone or in a rural area are shown in Table 1. The pre-existing underlying medical illness in these 157 patients included 14 (9.3%) patients having hypertension, 10 (6.7%) diabetes, 2 (1.3%) chronic renal disease, 2 (1.3%) chronic obstructive pulmonary disease, and 1 (0.7%) coronary heart disease. There was no significant difference between the mortality and survivor groups regarding their pre-existing underlying medical illness.

**Table 1:** Demographic data of the subjects who attempted suicide by paraquat self-poisoning.

Demographic variable	Suicide (n = 157)
Sex	
Male, n (%)	122 (77.7)
Female, n (%)	35 (22.3)
Age, mean ± SD (range)	41.2±14.9 (18–85)
Education, years*	9.3±3.1
Marital status, n (%)	
Single	44 (28.0)
Married	83 (52.9)
Divorced	20 (12.7)
Widowed	10 (6.4)
Jobless, n (%)	80 (51.0)
Living alone, n (%)	19 (12.1)

Of the 157 suicidal patients admitted to the nephrology ward through ER, 150 (94.9%) consented to a psychiatric consultation at the ward upon request from the medical staff, and our further analysis was centered on the remaining 150 patients. In these 150 patients, 45 (30%) patients reported previous suicide attempts or a history of self-harm, including 13 (8.7%) as paraquat suicide repeaters. The psychiatric diagnoses of the 150 patients are listed in table 2. It is worthy of note that several patients were of dual diagnosis. 1 patient met the criteria for a concurrent diagnosis of major depressive disorder and dysthymic disorder (i.e., double depression, defined as a major depressive disorder superimposing on an underlying dysthymic disorder) [29]. In patients diagnosed with substance use disorder, except for two having alcohol use disorder as their sole diagnosis, all had another axis 1 psychiatric comorbidities, including 14 concurrent major depressive disorders, 18 dysthymic disorders, 22 adjustment disorders, 2 psychotic disorders, and 1 bipolar disorder.

**Table 2:** Comorbid psychiatric diagnosis in the subjects who attempted suicide by paraquat self-poisoning.

Psychiatric diagnosis, n (%)	Suicide attempt withdiagnostic interview (n = 150)
Mood disorder	81 (54.0)
Major depressive disorder (MDD)†	38 (–25.3)
MDD With dysthymic disorder	1 (0.7)
Dysthymic disorder	40 (26.7)
Bipolar II disorder, depressed	4 (2.7)
Adjustment disorder	61 (40.7)
Schizophrenia or psychotic disorder, NOS	6 (4.0)
Substance use disorder$	59 (39.3)
Alcohol abuse or dependence	36 (24.0)
Illicit substance abuse or dependence	23 (15.3)
Heroin	5 (3.3)
Amphetamine	10 (6.7)
Heroin and amphetamine	6 (4.0)
Benzodiazepine	2 (1.3)

† One in the 38 patients diagnosed of major depressive disorder had concurrent dysthymic disorder.

$ Subjects with substance use disorder all had another axis 1 diagnosis, except for 2 patients of alcohol use disorder as sole diagnosis.

The mean score of the Social Readjustment Rating Scale is 66.9±4.8. Having a mental illness, couple conflict, and debt were the three major reasons for attempting suicide, the rest of which are listed in Table 3.

**Table 3:** Reasons for suicide attempt by paraquat intoxication.

Reasons	Number of patients, n (%)
Illness	54 (36.0)
Mental Illness	44 (29.3)
Physical Illness	10 (6.7)
Couple conflict	24 (16.0)
Debt	23 (15.3)
Parent-child conflict	16 (10.7)
Breaking up with boyfriend or girlfriend	11 (7.3)
Dismissal from work	8 (5.3)
Divorce	3 (2.0)
Job change	3 (2.0)
Death of parent	2 (1.3)
Legal problem	2 (1.3)
Examination	1 (0.7)
Financial status change	1 (0.7)
Conflict with parents-in-law	1 (0.7)
Pregnancy	1 (0.7)

Following paraquat ingestion, the 150 suicidal patients were brought to the emergency department after a mean of 4.0±9.2 hours, with a mean Glasgow coma scale score of 14.2±2.4 and mean serum paraquat level of 4.4±5.8 g/ml. A total of 16 (10.7%) and 39 patients (26.0%) developed hypotension and respiratory failure, respectively, and 87 patients (58.0%) died.

We further calculated the mortality risk by examining each demographic, psychiatric, or medical factor using univariate logistic regression analysis; our results are shown in Table 4. Age, hypotension, respiratory failure, acute renal failure, receiving pulse therapy, paraquat blood level, the amount of paraquat intake, a diagnosis of dysthymic disorder and depressive disorder (collectively including major depressive disorder, dysthymic disorder and bipolar depression) were significant variables associated with mortality. In each multivariate logistic regression model based on different psychiatric diagnoses (table 4 and tables S1–S7 in File S1), acute respiratory failure and SIPP were the two remaining significant medical predictors of mortality consistent throughout all models. Dysthymic disorder remained the only psychiatric diagnosis to independently predict mortality (OR = 5.58-, 95% CI: 1.13–27.69; p<0.05), even after controlling for other medical comorbidities, age and gender.

**Table 4:** Differences between paraquat suicide completers and survivors.

Variables	Mortality	Survivors	Univariate	Multivariate^a^
	(n = 87)	(n = 63)	odds ratio (95% CI)	odds ratio (95% CI)
Age	45.2±15.6	34.8±11.2	1.06 (1.03–1.09)***	-
Gender (male)	71 (81.6)	47 (74.6)	1.51 (0.69–3.31)	-
Living alone	13 (14.9)	6 (9.5)	1.67 (0.60–4.66)	-
Past suicide attempt	23 (36.5)	22 (25.3)	1.70 (0.94–3.44)	-
Concomitant alcohol use	22 (34.9)	21 (24.1)	0.57 (0.28–1.19)	-
Hypotension, n (%)	76 (87.4)	41 (65.1)	3.71 (1.64–8.40)**	-
Respiratory failure, n (%)	74 (85.1)	10 (15.9)	30.17 (12.31–73.96)***	-
Acute renal failure, n (%)	70 (80.5)	31 (49.2)	4.25 (2.06–8.77)***	-
Pulse therapy, n (%)	72 (82.8)	62 (98.4)	0.07 (0.01–0.60)*	-
Amount of paraquat intake, ml	97.9±68.5	65.6±42.1	1.01 (1.004–1.02)**	-
SIPP (h/mg/L)	30.4±40.5	2.7±3.4	1.21 (1.10–1.33)***	-
Paraquat blood level, g/mL	7.0±6.3	0.7±1.4	1.78 (1.39–2.28)***	-
Time between intake to ER (hr.)	10.7±18.8	17.5±25.6	0.99 (0.97–1.00)	-
Depressive disorder, n (%)	59 (72.8)	22 (27.2)	3.93 (1.98–7.80) ***	1.16 (0.31–4.34)
Major depressive disorder, n (%)	24 (27.6)	14 (22.2)	1.33 (0.63–2.84)	0.28 (0.05–1.47)
Dysthymic disorder, n (%)	35 (40.2)	5 (7.9)	7.81 (2.85–21.42)***	5.58 (1.13–27.69)*
Bipolar depression, n (%)	1 (1.1)	3 (4.8)	Not analyzed	Not analyzed
Schizophrenia or psychotic disorder	0	6 (9.5)	Not analyzed	Not analyzed
Adjustment disorder, n (%)	28 (32.2)	33 (52.4)	0.54 (0.28–1.05)	1.14 (0.30–4.28)
Substance use disorder, n (%)	35 (40.2)	24 (38.1)	1.01 (0.56–2.13)	2.77 (0.69–11.08)
Alcohol use disorder, n (%)	22 (25.3)	14 (22.2)	1.19 (0.55–2.55)	1.24 (0.29–5.26)
Illicit substance use disorder, n (%)	13 (14.9)	10 (15.9)	0.93 (0.38–2.28)	4.97 (0.73–33.99)

SIPP, severity index of paraquat poisoning.

a Multivariate analysis was calculate in each psychiatric diagnosis by calculating its odds ratio for mortality, controlling for age, gender, hypotension, acute renal failure, respiratory failure, pulse therapy, amount of paraquat intake, and SIPP.

*P<0.05, **p<0.01, ***p<0.001.

## Discussion

During the 10-year enrollment period, we found that the cause of paraquat poisoning in 87.8% of presenting patients was attributable to attempted suicide. This is similar to the nearly 90% rate in Japan [30] and the 73.4% rate in Korea [31]. Therefore, suicide prevention should be the focus in the prevention of paraquat poisoning.

In our study, 77.7% of overall patients are male. This connotes a gender ratio (male to female) of 3.5, which is higher than the 1.3 in Korea [31] and 1.2 in Sri Lank [32]. In the broader spectrum of pesticide suicide, China has been widely discussed for its female-predominant gender ratio [33], especially for rural young females [34]. Therefore, the gender ratio, even in the same method of suicide, differs region by region. In fact, our data echoes another national study in pesticide suicide in Taiwan (71.7% male prevalence) [35], and provides more representativeness in our sampling.

One common scenario in suicide attempts by paraquat self-poisoning is impulsive paraquat intake following family conflict [36]. In line with prior studies [4], [36], the precipitants of suicide in our study centered on intra-familial conflicts. Accordingly, it was postulated that the high fatality of pesticide suicide was due to its high lethality, which renders fatal even the attempts of those with low suicidality, and not due to inadequate treatment of mental illness [1]. Despite the identifiable stressors, one should not overlook the prevalence of mental illness in our subjects. Apart from adjustment disorder, more than half of the patients in the present study had a depressive disorder. In fact, mental illness, especially depressive disorder, has long been associated with suicide [37]. In addition, an increase in suicide rates was found to parallel an increase in mental illness over the past 20 years in Taiwan [38]. Early diagnosis and treatment of underlying mental illness is the key to suicide prevention.

Another important finding is that after adjusting for medical factors, dysthymic disorder was associated with a high risk of mortality. It is a well-established notion that depression increases mortality [39], [40], either through an increase in suicide rate [41] or via an unhealthy lifestyle, noncompliance with treatment, or dysregulation of the neuroendocrine system [42]–[44]. Our result could be explained partially by the hypothesis of depression as a risk factor for non-suicide mortality. However, the finding that only dysthymic disorder, but not major depressive disorder, posed an increased risk of mortality merits some elaboration. It has been found that nearly all dysthymic disorders harbored an episode of major depression for a period either before or after its index diagnosis [45]. Thus, dysthymic disorder should not be merely seen as a “milder” form of major depression. Dysthymic disorder and double depression should be regarded as the same chronic depression viewed at a different stage [45], with an even worse course than major depression alone [46]. Regarding the high prevalence of dysthymic disorder in current study, one of the possibilities was that many patients failed to fulfill the full criteria of major depression during long periods and would fall into the category of dysthymic disorder if their depression lasted more than 2 years. Besides, dysthymic disorder was specifically listed among all other diagnoses in the consultation special chart to be recorded during each consultation, and this would mitigate the presumed under-estimation of dysthymic disorder. As for the low double depression rate in our data, we suspect that it was due to a recall bias in the cross-sectional study whereby patients were more focused on their current suffering caused by the paraquat poisoning. Also, the lack of association of major depressive disorder and subsequent mortality in our study may stem from the inclusion of different subtypes of major depressive disorder. Another possibility came from our diagnostic approach in consultation. As depressive symptoms and medical illness interact bidirectionally, we adopted the inclusive approach, which was to include those depressive symptoms even when physical illness might be related to the symptoms [47]. Thus, in the diagnosis of major depression, especially for the current episode, this inclusive approach tended to over-diagnose and might possibly include those of adjustment disorder or demoralization [48]. However, the diagnosis of dysthymic disorder was exempt from the influence of current physical illness as the symptoms of depression must commence before the acute intoxication, otherwise they wouldn’t fit the 2-year duration criteria.

There was mounting evidence suggesting that chronic and non-chronic major depressive disorder may follow in a different trajectory [45]. Thus, more studies may be needed to prove whether chronic or non-chronic major depression exerts different risk on mortality. Since chronicity is the defining element of dysthymic disorder, our result reflects that chronic depression may confer a risk of mortality. Also, our results may provide additional support to the modification in DSM-V wherein persistent depressive disorder, including dysthymic disorder, chronic major depression and double depression, should be regarded as a distinct entity [49]–[51]. Although it is a well-established notion that substance use disorder increases the risk for suicide behavior [52], [53], as evidenced by its prevalence in our sample, our results fail to show its impact on subsequent mortality as dysthymic disorder. More studies are needed to clarify whether substance use disorder increases non-suicide mortality.

### Limitations and conclusion

There are several limitations worth noting. First, this was a retrospective study with inherent limitations. Second, our sample was derived only from one hospital and solely from inpatients transferred from the emergency department. Therefore, patients whose physical condition was not severe enough to require admission or who were dead on arrival were automatically excluded. Third, the inter-rater reliability of the consultant psychiatrists was not calculated. However, the chief of the consultation-liaison team (Y.Y. Juang) oversaw the quality and accuracy of all the diagnoses in the weekly meeting during the past 10-year study period. Also, some would argue that the physical discomfort of paraquat intoxication may jeopardize the psychiatric interview and affect validity of the diagnosis. For the very severe paraquat intoxication, in which multiple organ failure with initial coma led to ultimate mortality in a few hours to a few days [54], their psychiatric diagnosis relied more on the collateral information. However, most of our patients didn’t come in coma as the initial GCS score, averaged in 14.2±2.4, reflected their eligibility to receive psychiatric interview. As in moderate to severe intoxication, patients usually remain in normal consciousness until symptoms of respiratory impairment manifest over 3–7 days [54]. This gave our team adequate time to conduct routine consultation to ensure the validity of our diagnosis. In fact, in our experience, many patients didn’t comprehend the gravity of their situation until escalating respiratory distress appeared several days later. Lastly, it is difficult to generalize our result that dysthymic disorder aggravates the non-suicide mortality rate to other pesticides, as there are varying medical treatments specific to the different suicide agents to control for. Nevertheless, we chose paraquat among all other pesticides for its high lethality and for its thoroughly researched modalities of treatment in the past 50 years [14]. Thus, cooperating with experienced toxicologists is mandatory in this type of research as we delineated how mental illness affecting the outcome of mortality by controlling for medical aspects. As this was a retrospective cross-sectional study, we were unable to determine whether total lifetime duration of mental illness causing addictive detrimental effect. Also, more studies are needed to verify whether a causal link exists between paraquat exposure and subsequent depression, similar to that of organophosphate [55]. Longitudinal prospective studies to compare the mental status prior to and after chronic paraquat exposure may be required to answer this hypothesis.

In conclusion, faced with the rampant destruction paraquat unleashes on tens of thousands of lives worldwide, we cannot stress enough the importance of restricting access to paraquat [56]. In the practice of suicide prevention [57], primary care physicians and gatekeepers should be aware of chronic depressed patients’ accessibility to paraquat. On the other hand, those who have high accessibility to paraquat may get a screen for depression. A feasible prevention strategy is through public education toward their family members the need to monitor their mental status and the lethal consequences of paraquat poisoning, since nearly 80% of our subjects were not living alone. Should their family members have been equipped with this alertness, the ensuing tragedies could have been averted. Moreover, 30% of our patients are suicide repeaters and it merits even more attention from the public and the health professionals. This study reminds us the important of alertness to comorbid psychiatric illness, especially dysthymic disorder, in the prevention and treatment of suicide by paraquat poisoning.

## Supporting Information

File S1 **Supporting tables. Table S1**, Binary logistic regression (enter method) in the model of dysthymic disorder to predict the mortality of paraquat suicide, controlling for the medically related factors. **Table S2**, Binary logistic regression (enter method) in the model of depressive disorders to predict the mortality of paraquat suicide, controlling for the medically related factors. **Table S3**, Binary logistic regression (enter method) in the model of major depressive disorder to predict the mortality of paraquat suicide, controlling for the medically related factors. **Table S4**, Binary logistic regression (enter method) in the model of adjustment disorder to predict the mortality of paraquat suicide, controlling for the medically related factors. **Table S5**, Binary logistic regression (enter method) in the model of substance use disorder to predict the mortality of paraquat suicide, controlling for the medically related factors. **Table S6**, Binary logistic regression (enter method) in the model of alcohol use disorder to predict the mortality of paraquat suicide, controlling for the medically related factors. **Table S7**, Binary logistic regression (enter method) in the model of Illicit substance use disorder to predict the mortality of paraquat suicide, controlling for the medically related factors.
(DOCX)
