# Supplementary material for: Psychiatric Comorbidity and Its Impact on Mortality in Patients Who Attempted Suicide by Paraquat Poisoning during 2000–2010
Source: PLoS One. 2014 Nov 11;9(11):e112160. doi: 10.1371/journal.pone.0112160 (PMC4227688; doi:10.1371/journal.pone.0112160)
Supplement: File S1 — Supporting tables. Table S1, Binary logistic regression (enter method) in the model of dysthymic disorder to predict the mortality of paraquat suicide, controlling for the medically related factors. Table S2, Binary logistic regression (enter method) in the model of depressive disorders to predict the mortality of paraquat suicide, controlling for the medically related factors. Table S3, Binary logistic regression (enter method) in the model of major depressive disorder to predict the mortality of paraquat suicide, controlling for the medically related factors. Table S4, Binary logistic regression (enter method) in the model of adjustment disorder to predict the mortality of paraquat suicide, controlling for the medically related factors. Table S5, Binary logistic regression (enter method) in the model of substance use disorder to predict the mortality of paraquat suicide, controlling for the medically related factors. Table S6, Binary logistic regression (enter method) in the model of alcohol use disorder to predict the mortality of paraquat suicide, controlling for the medically related factors. Table S7, Binary logistic regression (enter method) in the model of Illicit substance use disorder to predict the mortality of paraquat suicide, controlling for the medically related factors. (DOCX) [file pone.0112160.s001.docx]

**Table S1. Binary logistic regression (enter method) in the model of dysthymic disorder to predict the mortality of paraquat suicide, controlling for the medically related factors.**

| Variables | B | Wald *X^2^* | Odds ratio^a^ | 95% CI | P value |
| --- | --- | --- | --- | --- | --- |
| Dysthymic disorder | 1.72 | 4.44 | 5.58 | 1.13-27.69 | 0.03* |
| Age | 0.03 | 0.89 | 1.03 | 0.97-1.10 | 0.35 |
| Male | 1.35 | 2.41 | 3.85 | 0.70-21.03 | 0.12 |
| Hypotension | 0.92 | 0.29 | 1.64 | 0.27-10.03 | 0.59 |
| Respiratory failure | 2.88 | 15.90 | 18.82 | 4.33-73.43 | 0.00** |
| Acute renal failure | 0.80 | 0.39 | 1.65 | 0.34-7.97 | 0.53 |
| Pulse therapy | -20.17 | 0.00 | 0.00 | 0.00 | 0.99 |
| SIPP (h/mg/L) | 0.15 | 4.25 | 1.16 | 1.01-1.33 | 0.04* |
| Amount of paraquat, ml | 0.01 | 0.99 | 1.01 | 0.99-1.02 | 0.32 |

^a^ Adjusted for all other variables in the fitted model.

* P < 0.05, ** p < 0.01, *** p < 0.001

**Table S2. Binary logistic regression (enter method) in the model of depressive disorders to predict the mortality of paraquat suicide, controlling for the medically related factors.**

| Variables | B | Wald *X^2^* | Odds ratio^a^ | 95% CI | P value |
| --- | --- | --- | --- | --- | --- |
| Depressive disorder | 0.15 | 0.05 | 1.16 | 0.31-4.34 | 0.83 |
| Age | 0.03 | 0.97 | 1.03 | 0.97-1.10 | 0.33 |
| Male | 1.66 | 3.91 | 5.24 | 1.01-27.11 | 0.05 |
| Hypotension | 0.48 | 0.31 | 1.62 | 0.30-8.81 | 0.58 |
| Respiratory failure | 2.92 | 16.86 | 18.52 | 4.60-74.60 | 0.00** |
| Acute renal failure | 0.57 | 0.54 | 1.77 | 0.39-8.06 | 0.46 |
| Pulse therapy | -21.10 | 0.00 | 0.00 | 0.00 | 0.99 |
| SIPP (h/mg/L) | 0.14 | 4.19 | 1.14 | 1.01-1.30 | 0.04* |
| Amount of paraquat, ml | 0.01 | 1.18 | 1.01 | 0.99-1.02 | 0.28 |

^a^ Adjusted for all other variables in the fitted model.

* P < 0.05, ** p < 0.01,

**Table S3. Binary logistic regression (enter method) in the model of major depressive disorder to predict the mortality of paraquat suicide, controlling for the medically related factors.**

| Variables | B | Wald *X^2^* | Odds ratio^a^ | 95% CI | P value |
| --- | --- | --- | --- | --- | --- |
| Major depressive disorder | -1.27 | 2.26 | 0.28 | 0.05-1.47 | 0.13 |
| Age | 0.04 | 1.44 | 1.04 | 0.98-1.11 | 0.23 |
| Male | 1.73 | 4.07 | 5.61 | 1.05-29.98 | 0.04* |
| Hypotension | 0.59 | 0.44 | 1.80 | 0.31-10.36 | 0.51 |
| Respiratory failure | 3.32 | 19.22 | 27.77 | 6.28-122.74 | 0.00** |
| Acute renal failure | 0.61 | 0.56 | 1.83 | 0.38-8.88 | 0.45 |
| Pulse therapy | -21.13 | 0.00 | 0.00 | 0.00 | 0.99 |
| SIPP (h/mg/L) | 0.15 | 3.92 | 1.16 | 1.01-1.35 | 0.04* |
| Amount of paraquat, ml | 0.01 | 1.45 | 1.01 | 0.99-1.02 | 0.23 |

^a^ Adjusted for all other variables in the fitted model.

* P < 0.05, ** p < 0.01, *** p < 0.001

**Table S4. Binary logistic regression (enter method) in the model of adjustment disorder to predict the mortality of paraquat suicide, controlling for the medically related factors.**

| Variables | B | Wald *X^2^* | Odds ratio^a^ | 95% CI | P value |
| --- | --- | --- | --- | --- | --- |
| Adjustment disorder | 0.13 | 0.04 | 1.14 | 0.30-4.28 | 0.85 |
| Age | 0.03 | 1.03 | 1.03 | 0.97-1.10 | 0.31 |
| Male | 1.67 | 3.955 | 5.28 | 1.02-27.20 | 0.05 |
| Hypotension | 0.51 | 0.34 | 1.66 | 0.31-8.97 | 0.56 |
| Respiratory failure | 2.99 | 18.57 | 19.87 | 5.10-77.40 | 0.00** |
| Acute renal failure | 0.60 | 0.60 | 1.82 | 0.40-8.38 | 0.44 |
| Pulse therapy | -21.26 | 0.00 | 0.00 | 0.00 | 0.99 |
| SIPP (h/mg/L) | 0.14 | 4.05 | 1.15 | 1.01-1.31 | 0.04* |
| Amount of paraquat, ml | 0.01 | 1.17 | 1.01 | 0.99-1.02 | 0.28 |

^a^ Adjusted for all other variables in the fitted model.

* P < 0.05, ** p < 0.01, *** p < 0.001

**Table S5. Binary logistic regression (enter method) in the model of substance use disorder to predict the mortality of paraquat suicide, controlling for the medically related factors.**

| Variables | B | Wald *X^2^* | Odds ratio^a^ | 95% CI | P value |
| --- | --- | --- | --- | --- | --- |
| Substance use disorder | 1.02 | 2.07 | 2.77 | 0.69-11.08 | 0.15 |
| Age | 0.04 | 1.13 | 1.04 | 0.97-1.10 | 0.29 |
| Male | 1.46 | 2.78 | 4.30 | 0.77-23.94 | 0.10 |
| Hypotension | 0.26 | 0.09 | 1.30 | 0.24-7.19 | 0.76 |
| Respiratory failure | 3.09 | 18.46 | 22.00 | 5.38-90.04 | 0.00*** |
| Acute renal failure | 0.55 | 0.47 | 1.73 | 0.36-8.21 | 0.49 |
| Pulse therapy | -20.79 | 0.00 | 0.00 | 0.00 | 0.99 |
| SIPP (h/mg/L) | 0.15 | 4.25 | 1.16 | 1.01-1.34 | 0.04* |
| Amount of paraquat, ml | 0.01 | 1.25 | 1.01 | 0.99-1.02 | 0.26 |

^a^ Adjusted for all other variables in the fitted model.

* P < 0.05, ** p < 0.01, *** p < 0.001

**Table S6. Binary logistic regression (enter method) in the model of alcohol use disorder to predict the mortality of paraquat suicide, controlling for the medically related factors.**

| Variables | B | Wald *X^2^* | Odds ratio^a^ | 95% CI | P value |
| --- | --- | --- | --- | --- | --- |
| Alcohol use disorder | 0.21 | 0.08 | 1.24 | 0.29-5.26 | 0.77 |
| Age | 0.03 | 0.97 | 1.03 | 0.97-1.10 | 0.33 |
| Male | 1.65 | 3.88 | 5.23 | 1.01-27.09 | 0.05 |
| Hypotension | 0.46 | 0.28 | 1.58 | 0.29-8.62 | 0.60 |
| Respiratory failure | 2.95 | 18.59 | 19.04 | 4.99-72.65 | 0.00** |
| Acute renal failure | 0.61 | 0.61 | 1.84 | 0.40-8.51 | 0.44 |
| Pulse therapy | -21.06 | 0.00 | 0.00 | 0.00 | 0.99 |
| SIPP (h/mg/L) | 0.14 | 4.14 | 1.15 | 1.01-1.31 | 0.04* |
| Amount of paraquat, ml | 0.01 | 1.17 | 1.01 | 0.99-1.02 | 0.28 |

^a^ Adjusted for all other variables in the fitted model.

* P < 0.05, ** p < 0.01, *** p < 0.001

**Table S7. Binary logistic regression (enter method) in the model of Illicit substance use disorder to predict the mortality of paraquat suicide, controlling for the medically related factors.**

| Variables | B | Wald *X^2^* | Odds  ratio^a^ | 95% CI | P value | |
| --- | --- | --- | --- | --- | --- | --- |
| Illicit substance use disorder | 1.60 | 2.67 | 4.97 | 0.73-33.99 | 0.10 |  |
| Age | 0.04 | 1.48 | 1.01 | 0.99-1.02 | 0.23 |  |
| Male | 1.48 | 3.00 | 4.41 | 0.82-23.62 | 0.08 |  |
| Hypotension | 0.38 | 0.18 | 1.47 | 0.25-8.57 | 0.67 |  |
| Respiratory failure | 3.34 | 18.53 | 28.13 | 6.16-128.50 | 0.00** |  |
| Acute renal failure | 0.52 | 0.45 | 1.69 | 0.36-7.85 | 0.50 |  |
| Pulse therapy | -21.38 | 0.00 | 0.00 | 0.00 | 0.99 |  |
| SIPP (h/mg/L) | 0.15 | 4.15 | 1.16 | 1.01-1.31 | 0.04* |  |
| Amount of paraquat, ml | 0.01 | 1.45 | 1.001 | 0.99-1.02 | 0.23 |  |

^a^ Adjusted for all other variables in the fitted model.

* P < 0.05, ** p < 0.01, *** p < 0.001
